# 4235 The Use of Checklists Throughout the Lifecourse of a Clinical Research Study: The Rockefeller University Checklist Suite

**DOI:** 10.1017/cts.2020.227

**Published:** 2020-07-29

**Authors:** Donna Brassil, Roger Vaughan, Arlene Hurley, Kathleen Dowd, Richard Hutt, Barry S. Coller

**Affiliations:** 1 Rockefeller University

## Abstract

OBJECTIVES/GOALS: We have developed a comprehensive Translational Research Navigation Program to guide investigators all the way from protocol development through study closure. As the program evolved, we initially developed organizational tools and then restructured them into a series of checklists to ensure that critical elements were not excluded or duplicated. METHODS/STUDY POPULATION: A series of checklists to assure that all research elements, including regulatory, scientific, and institutional, are addressed from protocol inception through study closure were developed by clinical research coordinators/navigators. The checklists are periodically updated and modified to reflect changing local and national regulations and policies. The first tool became the “Protocol Development Checklist” and then additional tools were developed and modified into a suite of navigation checklists that include “Protocol Implementation Checklist,” “Protocol Conduct Checklist,” and “Protocol Completion Checklist.” RESULTS/ANTICIPATED RESULTS: The checklists have been incorporated into the Translational Research Navigation Program and have enhanced the organization and quality of protocols throughout their lifespan. For example, implementation of the Protocol Development Checklist resulted in a reduction in time to IRB approval (currently 10 days), and implementation of the Protocol Implementation Checklist has impacted the time from IRB approval to study start-up. The Protocol Conduct Checklist has aided investigators in being better prepared and more organized for study conduct activities and the Protocol Closure Checklist has assured timely protocol closure and regulatory compliance, including reporting to ClinicalTrials.gov. DISCUSSION/SIGNIFICANCE OF IMPACT: Protocol checklists are powerful tools to enhance thoroughness, organization, and quality of the clinical research process. The Rockefeller University protocol checklists are available to the CTSA and Scientific Communities. CONFLICT OF INTEREST DESCRIPTION: NA.

